# Association of *CD14 -260 *(*-159*) *C>T *and asthma: a systematic review and meta-analysis

**DOI:** 10.1186/1471-2350-12-93

**Published:** 2011-07-11

**Authors:** Linlu Zhao, Michael B Bracken

**Affiliations:** 1 Center for Perinatal, Pediatric and Environmental Epidemiology, Yale School of Public Health, New Haven, Connecticut, USA

## Abstract

**Background:**

Asthma is a phenotypically diverse disease with genetic susceptibility. A single nucleotide polymorphism (SNP) in the *CD14 *gene at position *-260 *(also known as *-159*) *C>T *has been inconsistently associated with asthma. The aim of this study was to estimate the combined likelihood of developing asthma given the *CD14 -260C>T *genotype.

**Methods:**

Following the Preferred Reporting Items for Systematic Reviews and Meta-Analyses (PRISMA) guidelines, a systematic search and meta-analysis of the literature was conducted to estimate the association between this SNP and asthma. Planned subgroup analyses were performed to detect potential sources of heterogeneity from selected study characteristics. Post-hoc sensitivity analysis was performed to identify studies exerting excessive influence on among-study heterogeneity and combined effects.

**Results:**

Meta-analysis of 23 studies yielded a non-significant overall association with high heterogeneity across studies. After restricting analysis to studies using atopic asthma and non-atopic non-asthma case-control phenotypes and excluding studies influencing heterogeneity, the genotype-specific odds ratios (ORs) suggested a codominant model. Carriers of the TT and CT genotypes were about 33% less likely (OR = 0.67, 95% CI: 0.54-0.84) and about 20% less likely (OR = 0.80, 95% CI: 0.66-0.95), respectively, to have atopic asthma compared to carriers of the CC genotype. Among-study heterogeneity may be explained by overly broad asthma phenotype definitions, gene-environment interactions, and gene-gene interactions.

**Conclusions:**

A protective dose-response relationship between the *CD14 -260T *allele and atopic asthma susceptibility was observed. These results demonstrate the importance of precisely specified case-control groups as well as the need to assess interactions in the investigation of complex diseases such as asthma.

## Background

Asthma is a common, complex, chronic medical condition characterized by lung inflammation, reversible airflow obstruction, and enhanced airway responsiveness to a variety of environmental stimuli. Epidemiological evidence suggests increased asthma prevalence in recent decades with reduced international differences in asthma prevalence [1]. The most common asthma phenotype is atopic asthma, accounting for 56% of asthma cases in the United States [2]. Atopic asthma is an immunoglobulin E mediated hypersensitivity reaction triggered by environmental allergens, such as endotoxin and aero-allergens [3]. Although environmental factors are important determinants of asthma, numerous studies have revealed that asthma has a strong genetic component. Susceptibility genes have been identified from linkage, candidate gene association, and genome-wide association studies. As of 2010, over 250 different genes have been associated with asthma, including cluster of differentiation 14 (*CD14*) [4,5].

A well studied common single nucleotide polymorphism (SNP) in the promoter region of *CD14*, *-260C>T *(rs2569190; also reported as *CD14 -159*), is the focus of this review. *CD14 *encodes a receptor protein that binds to lipopolysaccharide (LPS), its primary ligand, and interacts with co-receptors toll-like receptor 4 (*TLR4*) and lymphocyte antigen 96 (*LY96*). CD14 is expressed on the surface of monocytes, macrophages, and neutrophils as membrane CD14 and in the serum as soluble CD14 and its expression may be partially regulated at the genetic level [6]. LPS, a principle component of endotoxin, induces lung inflammation and originates from the outer membrane of Gram-negative bacteria. Ligand binding activates innate immune system pathways that may trigger atopic asthma [7]. Atopic asthmatic subjects are more sensitive to respirable endotoxin than non-asthmatic subjects [8] and also show increased expression of CD14 after acute allergen provocation [9] and LPS inhalation [10].

Two earlier meta-analyses found an overall null association between the *CD14 -260C>T *polymorphism and asthma, where no association was reported in some studies and the risk variant identified as either the T or C allele in others [11,12]. Unfortunately, these meta-analyses lacked adequate reporting of methodology and included studies examining non-asthma phenotypes. A more recent meta-analysis found a significant decreased atopic asthma risk for the TT and CT genotypes compared with the CC genotype when analysis was restricted to studies of Asian populations and children [13]. However, that review had several significant errors regarding study inclusion, data abstraction, and analyses.

Due to the inconsistency of past meta-analyses, an updated review was conducted to estimate the meta-odds of developing asthma given the *-260C>T *genotype in *CD14*. Subgroup analyses were planned in order to explore potential sources of among-study heterogeneity by examining the effect of selected study characteristics on the combined effect estimate. Methodological issues in the literature studying this association are discussed.

## Methods

### Identification of eligible studies

Complete details of study methods are in Additional file 1. The review process followed the Preferred Reporting Items for Systematic Reviews and Meta-Analyses (PRISMA) guidelines [14]. A PubMed, EMBASE, and Scopus search was conducted on April 29, 2011 using a sensitive strategy to identify relevant articles. The HuGE Literature Finder database was consulted for its listing of articles under the asthma phenotype and *CD14*. An article in press at time of search was added to the review [4]. Reference lists of articles retained for review and past meta-analyses were inspected for relevant publications. No publication date or language restrictions were imposed.

Article titles and abstracts of studies identified from the searches were screened and excluded from further analysis for the following reasons: ineligible phenotype, ineligible SNP, review article, basic science research, or animal research. The full-text of studies passing initial screening was reviewed and excluded based on the aforementioned and following criteria: not case-control or nested case-control study design, unreported genotype frequencies, or subjects included in another study. Studies must have an asthma outcome definition that followed accepted diagnostic guidelines, was physician diagnosed, or used a combination of questionnaire and clinical ascertainment.

For multiple publications based on related data sets, the study with the greatest number of subjects was included. Reviewers extracted study information independently and disagreements were resolved by discussion and consensus.

### Statistical analysis

The general approach to meta-analysis has been described previously [15,16]. The pooled frequency of the putative risk allele (*-260T*) was estimated in various ethnic groups using the inverse variance method. Heterogeneity of studies was assessed using the *I^2 ^*statistic [17] separately for the genotype-specific odds ratios (ORs) across studies: TT versus CC (OR_1_), CT versus CC (OR_2_), and TT versus CT (OR_3_). If no or low heterogeneity existed (*I^2 ^*< 25%), the inverse variance method was used to estimate the pooled OR and 95% confidence interval (CI), assuming a fixed effects model. Otherwise, a random effects model was used. Comparisons of OR_1_, OR_2_, and OR_3 _indicated the most appropriate genetic model for the *-260T *allele [16].

Subgroup analyses were planned when sufficient information was reported in at least four studies in each subgroup. The effect of having more homogeneous case and control phenotype definitions (atopic asthma versus non-atopic non-asthma), ethnicity, age, publication year, or study size on the association was examined to identify potential sources of heterogeneity. Post-hoc sensitivity analysis using the sequential algorithm [18] with an *I^2 ^*threshold of 25% was conducted in the presence of significant among-study heterogeneity to evaluate studies responsible for the heterogeneity. Influence analysis was conducted to allow identification of studies excessively perturbing the summary estimate. Publication bias was assessed visually using a funnel plot of the standard error of the logarithm of the effect estimate against the effect estimate of each study.

Review Manager Version 5.1.1 (Nordic Cochrane Centre, Cochrane Collaboration, Copenhagen, Sweden) was used to conduct the meta-analysis, sequential analysis, and publication bias assessment. MetaAnalyst Version Beta 3.13 (Tufts Medical Center, Boston, MA) was used to estimate the pooled *-260T *allele frequency and conduct the influence analysis.

## Results

### Study inclusion and characteristics

**Figure 1:**
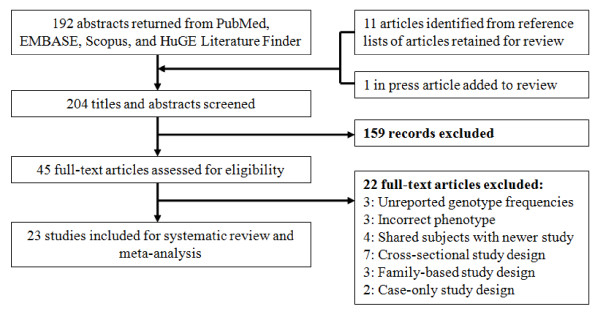
**Flow diagram of the systematic review and meta-analysis literature search results**. HuGE is the Human Genome Epidemiology Network.

The literature search identified 204 potentially relevant articles. Initial screening of titles and abstracts excluded 159 studies which did not meet the eligibility criteria. The full-text of the remaining 45 studies was retrieved for review: 22 additional studies were excluded. Unpublished *CD14 -260C>T *SNP data was provided by the corresponding author for one study [4]. Multiple publications were discovered for two data sets [19-23]. The studies with the largest number of subjects were retained [20,23]. Since Chan et al. [20] did not include genotype frequency data on atopic asthma cases and corresponding controls, this information was abstracted from the related paper with shared subjects by Leung et al. [21]. In total, this review yielded 23 studies [4,11,20,23-42] for meta-analysis. Two studies were published in Chinese [25,26] and one in Polish [32]. The search results revealed that it was necessary to search more than one database in order to capture all relevant studies. Figure 1 provides a summary of the search results.

All studies retained for review used either a case-control or nested case-control design. Of the 23 studies, 15 included mixed asthma cases [11,20,23,25,29,30,33-40,42], of which five separated asthma cases by atopic status [11,20,36,37,42], and eight included only atopic asthma cases [4,24,26-28,31,32,41]. Thirteen studies investigated European populations [4,11,24,27-36], eight investigated East Asian populations [20,23,25,26,37-40], and two investigated other populations [41,42]. Appropriate diagnostic criteria and proper genotyping methods were used in all studies. Eight studies applied some form of genotyping quality control and only two reported that genotyping was blinded to case-control status. Deviation from Hardy-Weinberg equilibrium (HWE) was detected in the controls of three studies [27,33,42]. Genotype frequencies for the studies by Bjornvold et al. [24] and Hakonarson et al. [28] could not be ascertained and were estimated based on reported allele frequencies, assuming HWE. All studies used unique samples: a total of 4780 genotyped asthma cases and 5650 genotyped non-asthmatic controls were included in the meta-analysis. Study characteristics and genotype frequencies are summarized in Table 1 (see Table S1, Additional file 2, for a complete summary of abstracted study characteristics).

**Table 1:** Characteristics and genotype distributions of reviewed studies on *CD14 -260 *(*-159*) *C>T *and asthma.

Study	Country	Study design	Outcome	Cases				Controls				
				
					Genotypes				Genotypes			
								
				N	CC	CT	TT	N	CC	CT	TT	HWE *p*
European												
Bjornvold [24]^*a*^	Norway	CC	AA	103	39	49	15	479	161	233	85	-
de Faria [27]	Brazil	CC	AA	88	27	41	20	202	63	131	8	< 0.01
Hakonarson [28]^*a*^	Iceland	CC	AA	94	31	46	17	94	29	46	19	-
Heinzmann [29]	Germany	CC	MA	182	51	89	42	261	79	124	58	0.48
Kedda [11]^*b, c*^	Australia	CC	AA, NAA	568	148	284	136	443	124	226	93	0.59
Koppelman [30]	Netherlands	CC	MA	159	51	76	32	158	31	85	42	0.31
Kowal [31]	Poland	CC	AA	372	141	152	79	160	42	73	45	0.27
Lis [32]	Poland	CC	AA	50	20	24	6	73	28	34	11	0.90
Murk [4]	USA	CC	AA	97	31	55	11	473	137	236	100	0.93
Sengler [33]	Germany	NCC	MA	84	23	43	18	119	26	72	21	0.02
Smit [34]	Denmark	NCC	MA	100	34	47	19	88	26	47	15	0.42
Smit [35]	France	CC	MA	223	49	107	67	554	145	276	133	0.94
Woo [36]^*b*^	USA	CC	AA, NAA	175	46	94	35	61	20	35	6	0.10
*Subtotal*				*2295*	*691*	*1107*	*497*	*3165*	*911*	*1618*	*636*	
East Asian												
Chan [20]^*d*^	Hong Kong	CC	MA	269	55	134	80	141	26	77	38	0.23
Chen [25]	China	CC	MA	150	63	62	25	150	40	68	42	0.25
Cui [26]	China	CC	AA	143	27	67	49	72	10	42	20	0.11
Hong [37]^*b*^	South Korea	CC	AA, NAA	626	113	284	229	153	22	71	60	0.89
Kuo Chou [38]	Taiwan	CC	MA	116	17	64	35	232	45	118	69	0.67
Park [39]	South Korea	CC	MA	85	16	39	30	550	90	267	193	0.88
Wang [23]	Taiwan	CC	MA	447	57	230	160	509	96	236	177	0.27
Wu [40]	China	CC	MA	252	54	117	81	227	31	121	75	0.10
*Subtotal*				*2088*	*402*	*997*	*689*	*2034*	*360*	*1000*	*674*	
Indian												
Sharma [41]	India	CC	AA	187	43	92	52	227	30	112	85	0.47
North African												
Lachheb [42]^*b*^	Tunisia	CC	AA, NAA	210	46	90	74	224	36	72	116	< 0.01
***Total***				***4780***	***1182***	***2286***	***1312***	***5650***	***1337***	***2802***	***1511***	

The number of successfully genotyped cases and controls may be less than the total number of cases and controls in the study (i.e. SNP call rate < 100%). Genotype frequencies presented as reported, otherwise calculated from reported genotype percent frequencies. Abbreviations: AA, atopic asthma; CC, case-control; HWE, Hardy-Weinberg equilibrium; MA, mixed asthma; N, genotyped sample size; NA, non-asthma; NAA, non-atopic asthma; NANA, non-atopic non-asthma; NCC, nested case-control.

*^a ^*Genotype frequencies estimated based on allele frequencies assuming HWE among cases and controls.

*^b ^*Genotype distribution for AA cases shown (genotype distribution for NAA cases not shown).

*^c ^*Genotype distribution for NA controls shown (genotype distribution for NANA controls not shown).

*^d ^*Genotype frequency information from this data set for atopic asthma cases and corresponding controls (not shown) abstracted from Leung et al. [21].

### Pooled *CD14 -260T *allele frequency in controls

Pooled *CD14 -260T *allele frequencies, using the inverse variance fixed effects model, were 0.457 (95% CI: 0.445-0.469) for overall European populations and 0.462 (95% CI: 0.449-0.475) for European populations excluding those not in HWE [27,33]. The pooled frequency was 0.577 (95% CI: 0.562-0.592) for East Asian populations. The *-260T *allele frequency was 0.621 (95% CI: 0.576-0.665) in an Indian population.

### Association between *CD14 -260C>T *and asthma risk

The pooled ORs for each pair-wise genotype comparison and corresponding *I^2 ^*statistics are summarized in Table 2. For all studies, heterogeneity ranged from moderate to high for the non-significant genotype-specific ORs, suggesting no association between the polymorphism and asthma risk. Subgroup analyses (data not shown) did not show significant gene effects when studies were subset by ethnicity (European or East Asian), age range of cases and controls (adults or children), year of study publication (2006-2010 or 2001-2005), and genotyped study sample size (≥ 100 cases and ≥ 100 controls or < 100 cases or < 100 controls). Low to moderate among-study heterogeneity was present in all subgroups for OR_2 _and moderate to high heterogeneity for OR_1 _and OR_3_. Sensitivity analysis excluding studies that appeared to account for appreciable heterogeneity and influence did not meaningfully change the results for overall and subgroup meta-analyses (data not shown). Relatively symmetrical funnel plots indicated the absence of publication bias for the genotype-specific ORs (see Figures S1-S3, Additional files 3, 4 and 5).

**Table 2:** Estimated ORs for *CD14 -260 *(*-159*) *C>T *and asthma.

	No. of studies	ORs (95% CI)			***I^2 ^*(%) ***^a^*			Suggested genetic model
			
		TT vs. CC (OR_1_)	CT vs. CC (OR_2_)	TT vs. CT (OR_3_)	OR_1_	OR_2_	OR_3_	
Overall	23	0.88 (0.70-1.10)	0.87 (0.76-1.00)	1.01 (0.86-1.19)	68	36	56	NS
								
AA cases and NANA controls	13	0.89 (0.63-1.25)	0.90 (0.77-1.05)	1.01 (0.75-1.35)	69	23	69	NS
	10 *^b^*	0.67 (0.54-0.84)	0.80 (0.66-0.95)	0.90 (0.75-1.08)	10	0	10	Codominant
European	8	1.11 (0.63-1.93)	0.97 (0.76-1.22)	1.14 (0.70-1.86)	79	36	75	NS
Children	8	0.92 (0.59-1.42)	0.89 (0.73-1.10)	1.05 (0.64-1.70)	64	0	80	NS
Year of publication								
2006-2010 *^c^*	7	0.86 (0.53-1.37)	0.84 (0.69-1.03)	0.98 (0.61-1.59)	73	0	81	NS
2001-2005	6	0.95 (0.56-1.61)	0.91 (0.65-1.28)	1.03 (0.81-1.31)	66	47	15	NS
No. of cases and controls *^d^*								
≥ 100 cases and ≥ 100 controls	6	0.73 (0.48-1.10)	0.88 (0.64-1.23)	0.83 (0.69-1.00)	70	64	22	NS
< 100 cases or < 100 controls	7	1.15 (0.63-2.07)	0.88 (0.70-1.12)	1.34 (0.72-2.49)	69	0	78	NS
								
NAA cases and NANA controls	5	0.88 (0.39-1.97)	1.02 (0.67-1.57)	0.83 (0.54-1.27)	78	38	43	NS

Abbreviations: AA, atopic asthma; NAA, non-atopic asthma; NANA, non-atopic non-asthma; NS, non-significant; OR, odds ratio.

*^a ^*Guideline for interpretation of the *I^2 ^*statistic: *I^2 ^*= 0% no heterogeneity, *I^2 ^*= 25% low heterogeneity, *I^2 ^*= 50% moderate heterogeneity, and *I^2 ^*= 75% high heterogeneity [17].

*^b ^*Excluding studies with excessive contribution to among-study heterogeneity identified by post-hoc sequential analysis [11,27,42].

*^c ^*Including the in press article at time of search by Murk et al. [4].

*^d ^*Numbers include only genotyped cases and controls.

### Subgroup analysis by case-control phenotype definitions

Initial subgroup analysis of studies that had defined case-control phenotypes as atopic asthma and non-atopic non-asthma showed non-significant gene effects plus high among-study heterogeneity (Table 2). Further subgrouping of studies comparing atopic asthmatics and non-atopic non-asthmatics by ethnicity (European only), age (children only), year of study publication, and genotyped study sample size did not meaningfully change the results. Post-hoc sensitivity analysis identified three studies that may be responsible for the significant among-study heterogeneity: Kedda et al. [11], de Faria et al. [27], and Lachheb et al. [42]. While reported study characteristics for these three studies were not atypical compared to other studies, the controls for the studies by de Faria et al. and Lachheb et al. deviated significantly from HWE (*p *< 0.01). Influence analysis found moderate influence on the combined effects exerted by these three studies.

**Figure 2:**
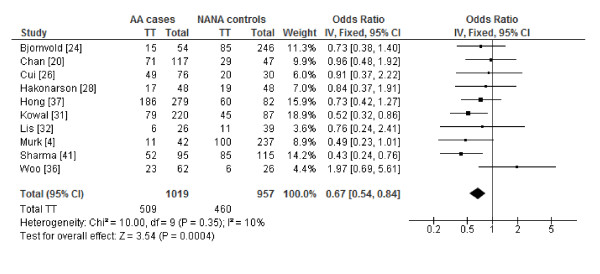
**Forest plot of *CD14 -260 *(*-159*) TT versus CC genotypes for studies with precisely defined phenotypes**. The forest plot displays the meta-analysis results of studies included in the review that used atopic asthma versus non-atopic non-asthma case-control phenotypes, excluding heterogeneous studies identified by sequential analysis [11,27,42]. Meta-analysis was conducted using an inverse variance (IV), fixed effects model. For each study in the forest plot, the area of the black square is proportional to study weight and the horizontal bar represents the 95% confidence interval (CI). Atopic asthma and non-atopic non-asthma are abbreviated as AA and NANA, respectively.

**Figure 3:**
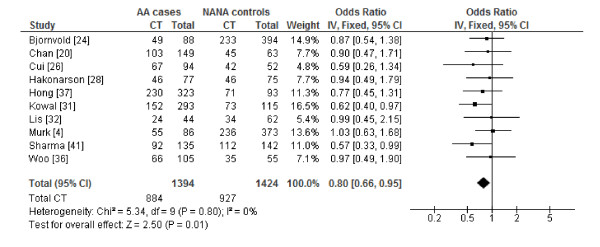
**Forest plot of *CD14 -260 *(*-159*) CT versus CC genotypes for studies with precisely defined phenotypes**. The forest plot displays the meta-analysis results of studies included in the review that used atopic asthma versus non-atopic non-asthma case-control phenotypes, excluding heterogeneous studies identified by sequential analysis [11,27,42]. Meta-analysis was conducted using an inverse variance (IV), fixed effects model. For each study in the forest plot, the area of the black square is proportional to study weight and the horizontal bar represents the 95% confidence interval (CI). Atopic asthma and non-atopic non-asthma are abbreviated as AA and NANA, respectively.

The genotype-specific ORs for the subgroup of studies with atopic asthma versus non-atopic non-asthma case-control groups, excluding the three studies identified by the post-hoc sequential analysis, implied a codominant model (Table 2). Compared to subjects with the CC genotype, the pooled ORs suggested that subjects with the TT genotype were some 33% less likely to have atopic asthma (OR_1 _= 0.67, 95% CI: 0.54-0.84, *I^2 ^*= 10%) (Figure 2) and subjects with the CT genotype were about 20% less likely to have atopic asthma (OR_2 _= 0.80, 95% CI: 0.66-0.95, *I^2 ^*= 0%) (Figure 3), showing a dose-response relationship for the T allele. No substantial heterogeneity was detected and publication bias was not evident in the funnel plots (see Figures S4 and S5, Additional files 6 and 7). Exclusion of any one particular study in the influence analysis did not meaningfully change the results (data not shown).

## Discussion

The present meta-analysis found a non-significant association between the *CD14 -260C>T *polymorphism and overall asthma. There was also high among-study heterogeneity in the meta-analysis, possibly accounting for the inconsistently reported findings between this SNP and asthma [43]. Subgroup analysis of selected study characteristics did not reveal any significant associations or substantial decreases in the *I^2 ^*estimate of heterogeneity. When restricting analysis to studies that used atopic asthma versus non-atopic non-asthma case-control phenotypes and excluding studies influencing heterogeneity, the genotype-specific ORs suggested a codominant model.

A sequential analysis revealed three studies that appeared to account for the high among-study heterogeneity (see Additional file 1 for methodology). Two had controls that departed from HWE, which may represent possible sources of bias. The exploratory nature of post-hoc sequential analysis may present a weakness, but advantages include its objective approach and the fact that specific study characteristics that may contribute to heterogeneity are not always known or recorded. The latter is important: if various methodological nuances are not reported, subsequent meta-analysis would not account for these factors and the ability to assess sources of heterogeneity would be hampered. For example, reported study characteristics in the article by Kedda et al. [11], one of the studies identified to incur a large amount of heterogeneity, did not reveal any particular characteristic that deviated from other studies.

Stronger associations and significant relationships were found when analysis was restricted to studies with more homogeneously defined case-control phenotypes and with heterogeneous studies excluded. These results indicated that the *-260T *allele was significantly protective under the codominant model when comparing atopic asthmatics to non-atopic non-asthmatics. Observed among-study heterogeneity may be partially explained by the employment of overly broad case-control phenotype definitions. It has been suggested in genome-wide association studies that use of homogeneous case phenotypes and precisely specified control groups—those who unambiguously do not have the case phenotype—may improve study efficiency [44]. This principle, borrowed from extreme discordant sib-pair analysis [45], naturally extends to case-control selection in candidate gene association studies.

There is possible gene-environment interaction, in which the SNP acts as a modifier of asthma risk in individuals with different degrees of environmental endotoxin exposure. Carriers of the TT genotype have been found to have higher serum levels of CD14 than carriers of the CT or CC genotypes [43]. This epidemiologic evidence is supported by functional genomic studies that showed increased transcriptional activity of the *-260T *allele in a monocytic cell line [46]. An antagonistic interaction has been demonstrated between *CD14 *and endotoxin exposure: homozygotes for the T allele appear to be protective for asthma at low levels of endotoxin exposure, but may increase asthma risk at high levels of endotoxin exposure [43]. Based on these findings, Martinez [43] hypothesized that higher CD14 expression in TT homozygotes increased sensitivity to the protective effects of low level endotoxin exposure compared to carriers of other genotypes. However, at higher levels of endotoxin exposure, induced CD14 expression could be increased in carriers of the C allele, showing a reversed protective effect. The findings of the present meta-analysis, restricted to studies using the atopic asthma versus non-atopic non-asthma case-control phenotypes, are consistent with this hypothesis at low endotoxin exposure levels. The codominant model for the *-260T *allele implied a dose-response relationship in CD14 expression and reduction of atopic asthma risk. This gene-environment interaction may be a source of heterogeneity among studies in the present and earlier meta-analyses [11-13].

In addition to the promoter, many additional regulatory elements are necessary to influence gene expression, particularly for genes like *CD14*, which exhibit highly complex expression patterns. Regulatory elements, such as enhancers and repressors, may reside in intronic regions or up- and down-stream of the transcriptional unit [47]. A risk variant with no obvious and no known function may regulate a gene at a considerable genomic distance from the location of the SNP. Therefore, it is important to study the influence of gene-gene interaction as well as other polymorphisms in *CD14 *on the effects of this locus on asthma susceptibility.

### Quality and methodology of studies

Assessing study quality was difficult due to inadequate reporting from all studies included in the meta-analysis. Many studies reported insufficient information about recruitment methodology and study participant characteristics, particularly for controls. Genotype distributions of controls departed from HWE in three studies [27,33,42]. Deviation from HWE in controls, or healthy populations, may indicate selection bias, population stratification, or genotyping errors [48]. Even in the absence of deviation from HWE, these biases could not be assessed given the inadequately reported information. Eight studies reported implementing some form of genotyping quality control [4,20,28,30,37,38,40,41]. Only two published studies mentioned blinding of phenotype when genotyping [29,38]. Furthermore, there is a potential for publication bias, where positive rather than negative findings tend to be published [49]. The completeness of evidence is also impeded by language bias. Studies conducted in non-English speaking countries tend to publish significant results in international journals and non-significant results in local journals, many of which are not indexed [50]. Selective publication of polymorphism and disease associations may obscure their true relationships.

Results from the pooled *CD14 -260T *allele frequency in controls revealed differences among the broad ethnic categories: 0.457 for European populations, 0.577 for East Asian populations, and 0.621 for an Indian population. In comparison, the International HapMap Project (Phase 3) reported the *-260T *allele frequency among Utah residents with Northern and Western European ancestry, Han Chinese in Beijing, China, Japanese in Tokyo, Japan, and Yoruba in Ibadan, Nigeria to be 0.474, 0.500, 0.488, and 0.293, respectively. The average heterozygosity reported in Build 132 of dbSNP is 0.488 ± 0.078 [51]. Interethnic differences in the allele frequencies of the *CD14 -260C>T *polymorphism is of concern as some studies included in this meta-analysis have different ethnic compositions between the cases and controls. Reported associations in studies of varying ethnic composition may have been influenced by population stratification. Even among apparently homogeneous ethnic groups, population stratification may be a problem [52]. The effect of this type of stratification has been reported to be small in most situations, but a small bias may be important in studies of genetic association, which typically consider small or moderate effects [53]. Only four studies included in this review reported an assessment of population stratification.

A commonly cited solution to addressing population stratification is the use of family-based designs to study genetic associations [44,54]. However, the family-based design has its own inherent limitation to susceptibility variant discovery. It has been argued that neither common nor rare genetic variants are heritable, as they do not give rise to a substantial familial concentration of cases due to low penetrance [55]. Three family-based studies have explored the association of *CD14 -260C>T *and asthma with conflicting results [12,41,42]. Therefore, efforts should be made to accrue controls from the same source population as cases to avoid population stratification, particularly when ethnicity is not matched or controlled [44].

## Conclusions

This meta-analysis provides a comprehensive examination of the available evidence concerning the association between the *CD14 -260C>T *polymorphism and asthma susceptibility. The significant association between this polymorphism and atopic asthma may be of clinical and public health importance. The genetics of asthma follow the "common disease, common variants" hypothesis, which posits that multiple genetic variants of interest are common to many individuals with the disease. These common variants typically have weak individual effects and low penetrance, but their high frequency confers a relatively large attributable risk in the population. Therefore, this common polymorphism, along with endotoxin exposure level information, has potential to be a useful and efficient predictor of atopic asthma risk. This review also emphasizes the importance of having precisely defined case-control groups to study complex diseases and demonstrates the need to incorporate gene-environment and gene-gene interaction analyses in future epidemiological investigations of asthma genetics.

## Competing interests

The authors declare that they have no competing interests.

## Authors' contributions

Both authors conceived and designed the study, performed the statistical analysis and interpretation, drafted the manuscript, revised for important intellectual content, and read and approved the final manuscript.

## Pre-publication history

The pre-publication history for this paper can be accessed here:

http://www.biomedcentral.com/1471-2350/12/93/prepub

## Supplementary Material

Additional file 1 **Supplemental methods**. Complete details of the study methodology. Click here for file

Additional file 2 **Table S1. Summary of abstracted characteristics of reviewed studies on *CD14 -260 *(*-159*) *C>T *and asthma**. Complete summary of abstracted characteristics from studies included in the systematic review and meta-analysis. Click here for file

Additional file 3 **Figure S1. Funnel plot of *CD14 -260 *(*-159*) TT versus CC genotypes for all reviewed studies**. Standard error of the logarithm of the odds ratio (SE(log[OR])) was plotted against the OR of each study. Click here for file

Additional file 4 **Figure S2. Funnel plot of *CD14 -260 *(*-159*) CT versus CC genotypes for all reviewed studies**. Standard error of the logarithm of the odds ratio (SE(log[OR])) was plotted against the OR of each study. Click here for file

Additional file 5 **Figure S3. Funnel plot of *CD14 -260 *(*-159*) TT versus CT genotypes for all reviewed studies**. Standard error of the logarithm of the odds ratio (SE(log[OR])) was plotted against the OR of each study. Click here for file

Additional file 6 **Figure S4. Funnel plot of *CD14 -260 *(*-159*) TT versus CC genotypes for studies with precisely defined phenotypes**. The funnel plot displays studies included in the review that used atopic asthma cases and non-atopic non-asthmatic controls, excluding heterogeneous studies identified by sequential analysis [11,27,42]. Standard error of the logarithm of the odds ratio (SE(log[OR])) was plotted against the OR of each study. Click here for file

Additional file 7 **Figure S5. Funnel plot of *CD14 -260 *(*-159*) CT versus CC genotypes for studies with precisely defined phenotypes**. The funnel plot displays studies included in the review that used atopic asthma cases and non-atopic non-asthmatic controls, excluding heterogeneous studies identified by sequential analysis [11,27,42]. Standard error of the logarithm of the odds ratio (SE(log[OR])) was plotted against the OR of each study. Click here for file
