# Supplementary material for: Association of CD14 -260 (-159) C>T and asthma: a systematic review and meta-analysis
Source: BMC Med Genet. 2011 Jul 11;12:93. doi: 10.1186/1471-2350-12-93 (PMC3148550; doi:10.1186/1471-2350-12-93)
Supplement: Additional file 1 — Supplemental methods. Complete details of the study methodology. [file 1471-2350-12-93-S1.DOC]

**SUPPLEMENTAL METHODS**

This systematic review and meta-analysis does not have a published review protocol and registration number. An unpublished protocol for internal comment was prepared using Review Manager Version 5.1.1 (Nordic Cochrane Centre, Cochrane Collaboration, Copenhagen, Sweden). The review process followed the Preferred Reporting Items for Systematic Reviews and Meta-Analyses (PRISMA) guidelines [1].

**Search Strategy**

A PubMed, EMBASE, and Scopus search was conducted on April 29, 2011 for articles whose title, abstract, or keywords contained “CD14,” “asthma,” and at least one of the following words: “polymorphism,” “mutation,” or “genotype.” The HuGE Literature Finder database was consulted for its listing of articles under the asthma phenotype and *CD14*. An article in press at time of search was added to the review [2]. Reference lists of articles retained for review and past meta-analyses were inspected for relevant publications not identified from the databases. No publication date or language restrictions were imposed.

**Study Selection**

Studies identified from the database searches and reference lists were loaded into RefWorks (ProQuest LLC, Ann Arbor, MI) and duplicates were removed. Article titles and abstracts were screened and excluded from further analysis for the following reasons: ineligible phenotype, ineligible SNP, review article, basic science research, or animal research. The full-text of studies passing initial screening was obtained and reviewed. Articles were excluded based on the aforementioned and following criteria: not case-control or nested case-control study design or subjects included in another study. Corresponding authors of otherwise eligible studies with unreported genotype frequencies were contacted and these studies were excluded if a response was not received within one month of initial contact. For multiple publications based on related data sets, the study with the greatest number of subjects was included.

Studies must have an asthma outcome definition that followed accepted diagnostic guidelines (e.g. American Thoracic Society criteria), was physician diagnosed, or used a combination of questionnaire and clinical ascertainment (e.g. pulmonary function test, bronchial hyperresponsiveness test).

**GWAS of Asthma**

A supplemental search for genome-wide association studies (GWAS) of asthma was conducted on April 29, 2011 using PubMed, EMBASE, and Scopus. Identified studies with eligible study designs and phenotype definitions did not use single nucleotide polymorphism (SNP) genotyping arrays that assayed the SNP of interest (*CD14 -260C>T*; rs2569190). Early and current generation SNP genotyping arrays have no or low SNP coverage within and surrounding the *CD14* locus [3]. Any studies that may have used the few current generation SNP arrays with rs2569190 assay (e.g. Illumina Human CytoSNP-12 and Omni1-Quad) have not been published at time of search. Since existing GWAS coverage for *CD14* is insufficient and imputation accuracy based on these data is inadequate [4], GWAS of asthma were not further pursued for inclusion in this systematic review and meta-analysis.

**Data Extraction**

The following information was extracted from each study included in the meta-analysis: first author’s last name, publication date, country where the study was conducted, case-control characteristics (number of subjects, type of asthma, age, gender, and ethnic composition), genotyping method and quality control, allele and genotype frequencies, and asthma diagnostic criteria. Reviewers extracted study information independently and disagreements were resolved by discussion and consensus. Reviewers were not blinded to names of authors, journals, and institutions.

**Statistical Analysis**

The general approach to meta-analysis has been described previously [5-6]. First, the chi-square (*χ^2^*) goodness of fit test was used to test the genotype distribution of controls of each study for deviation from Hardy-Weinberg equilibrium. Second, the pooled frequency of the putative risk allele (*-260T*) was estimated in various ethnic groups using the inverse variance method. Genotype distribution of the control group was used to estimate the T allele frequency when the latter was not reported. If ethnicity of participants was not reported, ethnicity was assumed according to the geographic location from which the participants were recruited. Third, the heterogeneity of studies was assessed using the *I^2^* statistic [7] separately for the genotype-specific odds ratios (ORs) across studies: TT versus CC (OR_1_), CT versus CC (OR_2_), and TT versus CT (OR_3_). If no or low heterogeneity existed (*I^2^* < 25%), the inverse variance method was used to estimate the pooled OR and 95% confidence interval, assuming a fixed effects model. Otherwise, a random effects model was used. Comparisons of OR_1_, OR_2_, and OR_3_ indicated the most appropriate genetic model for the *-260T* allele according to the following criteria [6]:

1. Recessive model if OR_1_ = OR_3_ ≠ 1 and OR_2_ = 1.
2. Dominant model if OR_1_ = OR_2_ ≠ 1 and OR_3_ = 1.
3. Overdominant model if OR_2_ = 1/OR_3_ ≠ 1 and OR_1_ = 1.
4. Codominant model if OR_1_ > OR_2_ > 1 and OR_1_ > OR_3_ > 1 (or OR_1_ < OR_2_ < 1 and OR_1_ < OR_3_ < 1).

Subgroup analyses were planned when sufficient information was reported in at least four studies in each subgroup. The effect of having more homogeneous case and control phenotype definitions (atopic asthma versus non-atopic non-asthma), ethnicity, age, publication year, or study size on the association was examined to identify potential sources of heterogeneity. Post-hoc sensitivity analysis was conducted in the presence of significant among-study heterogeneity to evaluate studies responsible for the heterogeneity. The sequential algorithm [8] was used with an *I^2^* threshold of 25%. Briefly, for *n* studies, one study was excluded from meta-analysis calculations at a time and the study responsible for the largest decrease in *I^2^* was removed. A new set of *n*-1 studies was created and the process repeated until the *I^2^* dropped below the desired threshold. A study that was removed from one genotype-specific OR was also removed from other genotype-specific OR calculations. Influence analysis was conducted to allow identification of studies excessively perturbing the summary estimate by recalculating the pooled estimate omitting one study at a time. Publication bias was assessed visually using a funnel plot of the standard error of the logarithm of the effect estimate against the effect estimate of each study.

Review Manager Version 5.1.1 was used to conduct the meta-analysis, sequential analysis, and publication bias assessment. MetaAnalyst Version Beta 3.13 (Tufts Medical Center, Boston, MA) was used to estimate the pooled *-260T* allele frequency and conduct the influence analysis.

**REFERENCES**

1. Moher D, Liberati A, Tetzlaff J, Altman DG, the PRISMA Group: **Preferred reporting items for systematic reviews and meta-analyses: the PRISMA statement.** *PLoS Med* 2009, **6**(7):e1000097.
2. Murk W, Walsh K, Hsu L-I, Zhao L, Bracken MB, DeWan AT: **Attempted replication of 50 reported asthma risk genes identifies SNPs in RAD50 and PTPRE as associated with childhood atopic asthma.** *Hum Hered*, in press.
3. Fujita PA, Rhead B, Zweig AS, Hinrichs AS, Karolchik D, Cline MS, Goldman M, Barber GP, Clawson H, Coelho A, Diekhans M, Dreszer TR, Giardine BM, Harte RA, Hillman-Jackson J, Hsu F, Kirkup V, Kuhn RM, Learned K, Li CH, Meyer LR, Pohl A, Raney BJ, Rosenbloom KR, Smith KE, Haussler D, Kent WJ: **The UCSC Genome Browser database: update 2011.** *Nucleic Acids Res* 2011, **39**(Suppl 1):D876-D882.
4. Michel S, Liang L, Depner M, Klopp N, Ruether A, Kumar A, Schedel M, Vogelberg C, von Mutius E, von Berg A, Bufe A, Rietschel E, Heinzmann A, Laub O, Simma B, Frischer T, Genuneit J, Gut IG, Schreiber S, Lathrop M, Illig T, Kabesch M: **Unifying candidate gene and GWAS Approaches in Asthma.** *PLoS One* 2010, **5**(11):e13894.
5. Zhang HF, Zhong BL, Zhu WL, Xie SL, Qiu LX, Zhu LG, Wang Y, Lei L: **CD14 C-260T gene polymorphism and ischemic heart disease susceptibility: a HuGE review and meta-analysis.** *Genet Med* 2009, **11**(6):403-408.
6. Thakkinstian A, McElduff P, D'Este C, Duffy D, Attia J: **A method for meta-analysis of molecular association studies.** *Stat Med* 2005, **24**(9):1291-1306.
7. Higgins JP, Thompson SG, Deeks JJ, Altman DG: **Measuring inconsistency in meta-analyses.** *BMJ* 2003, **327**(7414):557-560.
8. Patsopoulos NA, Evangelou E, Ioannidis JP: **Sensitivity of between-study heterogeneity in meta-analysis: proposed metrics and empirical evaluation.** *Int J Epidemiol* 2008, **37**(5):1148-1157.
