# Supplementary material for: Association of CD14 -260 (-159) C>T and asthma: a systematic review and meta-analysis
Source: BMC Med Genet. 2011 Jul 11;12:93. doi: 10.1186/1471-2350-12-93 (PMC3148550; doi:10.1186/1471-2350-12-93)
Supplement: Additional file 2 — Table S1. Summary of abstracted characteristics of reviewed studies on CD14 -260 (-159) C>T and asthma. Complete summary of abstracted characteristics from studies included in the systematic review and meta-analysis. [file 1471-2350-12-93-S2.DOC]

**Table S1. Summary of abstracted characteristics of reviewed studies on *CD14 -260* (*-159*) *C>T* and asthma**

Abbreviations: ATS, American Thoracic Society; BHR, bronchial hyperresponsiveness; GINA, Global Initiative for Asthma; IgE, immunoglobulin E; PCR-CE, polymerase chain reaction-capillary electrophoresis; PCR-FRET, polymerase chain reaction-fluorescence resonance energy transfer; PCR-RFLP, polymerase chain reaction-restriction fragment length polymorphism; PCR-SSP, polymerase chain reaction-sequence-specific primers; SNP, single nucleotide polymorphism; SPT, skin prick test.

| **Study** | **SNP detection and confirmation** | **No. of cases** | **Description of cases** | **No. of controls** | **Description of controls** | **Asthma and/or atopic asthma diagnostic criteria** |
| --- | --- | --- | --- | --- | --- | --- |
| Bjornvold [24] | PCR-CE | 108 | Atopic asthmatic children; 75% male; >95% Norwegian ethnicity | 496 | Non-atopic non-asthmatic children; 54% male; >95% Norwegian ethnicity | Atopic asthma diagnosed by positive SPT and having two of the following: (1) symptoms, (2) medication, and (3) doctor’s diagnosis by age 10. |
| Chan [20] *^a^* | PCR-RFLP; DNA sequencing (30 randomly selected samples) | 298 | Asthmatic children; 65% male; Chinese ethnicity | 175 | Children who did not have allergic or immunological disease; 55% male; Chinese ethnicity | Asthma was diagnosed according to ATS criteria. |
| Chen [25] | PCR-RFLP | 150 | Asthmatic children and adults; 52% male; Chinese ethnicity | 15 | Non-atopic non-asthmatic children and adults; 61% male; Chinese ethnicity | Asthma was diagnosed according to Chinese Society of Respiratory Diseases guidelines. |
| Cui [26] | PCR-RFLP | 143 | Atopic asthmatic children; 51% male; Chinese ethnicity | 72 | Non-atopic non-asthmatic children; 53% male; Chinese ethnicity | Asthma was physician diagnosed. Atopic status was established by total serum IgE level. |
| de Faria [27] | PCR-RFLP | 88 | Atopic asthmatic children; 47% male; 92% Caucasian, 7% mulatto, and 1% black ethnicity | 202 | Non-atopic non-asthmatic adults; 79% Caucasian, 20% black, and <1% other ethnicity | Asthma was diagnosed according to GINA criteria. Atopic status was diagnosed by positive SPT and total serum IgE level. |
| Hakonarson [28] | DNA sequencing | 94 | Atopic asthmatic children and adults; 40% male | 94 | Non-atopic non-asthmatic children and adults; 40% male | Asthma was diagnosed according to ATS criteria. Atopic status was diagnosed by positive SPT and total serum IgE level. |
| Heinzmann [29] | PCR-RFLP | 182 | Asthmatic children; 65% male; Caucasian ethnicity | 270 | Undiagnosed asthma adult probands; 60% male | Asthma was diagnosed by patient history of asthmatic symptoms, use of anti-asthmatic medication, and presence of some degree of BHR. Diagnosis was confirmed by standard pulmonary function tests and histamine challenge. |
| Hong [37] | PCR-RFLP; DNA sequencing for 20% subjects (randomly selected) | 635 | 635 asthmatic children (518 atopic asthmatics, 117 non-atopic asthmatics); 64% male; Korean ethnicity | 153 | Non-atopic non-asthmatic children; 53% male; Korean ethnicity | Asthma was diagnosed according to ATS criteria. Atopic status was diagnosed by positive SPT and increased allergen-specific serum IgE level. |
| Kedda [12] | PCR-RFLP | 568 | Asthmatic adults (472 atopic asthmatics, 96 non-atopic asthmatics); Caucasian ethnicity | 443 | Non-asthmatic adults (226 non-atopic non-asthmatics); Caucasian ethnicity | Asthma cases were physician diagnosed and confirmed by lung function tests. Atopic status was diagnosed by positive SPT. |
| Koppelman [30] | PCR-RFLP; DNA sequencing (32 cases and controls) | 159 | Asthmatic adults; 64% male; Caucasian ethnicity | 158 | Spouses of probands (14 asthmatics); 37% male; Caucasian ethnicity | Asthma cases were physician diagnosed and confirmed by lung function tests. Asthma was considered present in the spouses if they reported being under current regular treatment for asthma or under a physician’s care for asthma treatment. |
| Kowal [31] | PCR-RFLP | 372 | Atopic asthmatic adults; 57% male; white Polish ethnicity | 160 | Non-atopic non-asthmatic adults; 60% male; white Polish ethnicity | Asthma status was confirmed by lung function tests. Atopic status was diagnosed by positive SPT and *Dermatophagoides pteronyssinus*-specific serum IgE level. |
| Kuo Chou [38] | PCR-RFLP; assay repeated for 20% subjects (randomly selected) | 116 | Asthmatic children; 66% male; Chinese ethnicity | 232 | Non-asthmatic children; 66% male; Chinese ethnicity | Asthma was diagnosed according to ATS criteria. |
| Lachheb [42] | PCR-RFLP | 210 | Asthmatic children (139 atopic asthmatics, 71 non-atopic asthmatics); 66% male; white African ethnicity | 224 | Non-atopic non-asthmatic children; 57% male; white African ethnicity | Asthma was diagnosed according to GINA guidelines. Atopic status was diagnosed by positive SPT and total serum IgE level. |
| Lis [32] | PCR-RFLP | 50 | Atopic asthmatic children; 66% male | 73 | Non-atopic non-asthmatic children; 64% male | Asthma status was determined by self-reported positive history of asthma-like symptoms in the last year or physician diagnosis. Atopic status was confirmed by total serum IgE level. |
| Murk [4] | TaqMan genotyping | 103 | Atopic asthmatic children; 63% male; 43% Caucasian, 24% Hispanic, 20% biracial, 12% black, and <1% other | 499 | Non-atopic non-asthmatic children; 46% male; 76% Caucasian, 8% Hispanic, 8% biracial, 6% black, and <1% other | Atopic asthma cases were physician-diagnosed with wheezing at ages one and six years, asthma medication use, and allergies as reported by the mother. |
| Park [39] | PCR-RFLP | 85 | Asthmatic children and adults | 550 | Non-asthmatic subjects | Asthma cases were diagnosed on the basis of airway reversibility with methacholine challenge. |
| Sengler [33] | PCR-RFLP or PCR-FRET | 84 | Asthmatic children; Caucasian ethnicity | 119 | Non-atopic non-asthmatic children; Caucasian ethnicity | Asthma was diagnosed as one or more wheezing episodes during the previous 12 months. |
| Sharma [41] | PCR-RFLP; DNA sequencing (20 cases and 20 controls) | 187 | Atopic asthmatic children and adults; Indo-Aryan ethnicity | 227 | Non-atopic non-asthmatic children and adults; Indo-Aryan ethnicity | Asthma was diagnosed according to ATS criteria. Atopic status was established by self-reported history and total serum IgE level. |
| Smit [34] | PCR-SSP | 100 | Asthmatic children and adults; 85% male | 88 | Non-asthmatic children and adults; 90% male | Asthma was diagnosed based on study-specific survey. |
| Smit [35] | TaqMan genotyping | 239 | Asthmatic adults; 52% male | 586 | Non-asthmatic adults; 50% male | Asthma was diagnosed based on study-specific survey. |
| Wang [23] | TaqMan genotyping | 449 | Asthmatic children; 67% male | 512 | Non-asthmatic children; 48% male | Asthma was diagnosed by a modified British Medical Society respiratory questionnaire and confirmed by lung function testing. |
| Woo [36] | PCR-RFLP | 175 | Asthmatic children and adults (128 atopic asthmatics, 47 non-atopic asthmatics); 34% male; 82% Caucasian, 17% black, and 1% unknown ethnicity (also reported genotypes for Caucasian only) | 61 | Non-atopic non-asthmatic adults; 53% male; 80% Caucasian, 10% other, 7% unknown, and 3% black ethnicity | Asthma was confirmed according to ATS criteria. Atopic status was confirmed by positive SPT. |
| Wu [40] | PCR-RFLP; DNA sequencing (20 randomly selected subjects) | 252 | Asthmatic children; 55% male; Chinese ethnicity | 227 | Non-atopic non-asthmatic children; 52% male; Chinese ethnicity | Asthma was confirmed according to ATS criteria. |

*^a^* Genotype frequency information from this data set for atopic asthma cases and corresponding controls was abstracted from Leung et al. [21]. This subset from 2003 included 220 atopic asthmatic children and 92 children who did not have allergic or immunological disease. The proportions of males among cases and controls were 64% and 55%, respectively. All subjects were of Chinese ethnicity. Asthma was diagnosed according to ATS criteria and atopic status was diagnosed by the presence of at least one allergen-specific IgE in serum.
