# Supplementary figures and images for: Association of CD14 -260 (-159) C>T and asthma: a systematic review and meta-analysis
Source: BMC Med Genet. 2011 Jul 11;12:93. doi: 10.1186/1471-2350-12-93 (PMC3148550; doi:10.1186/1471-2350-12-93)

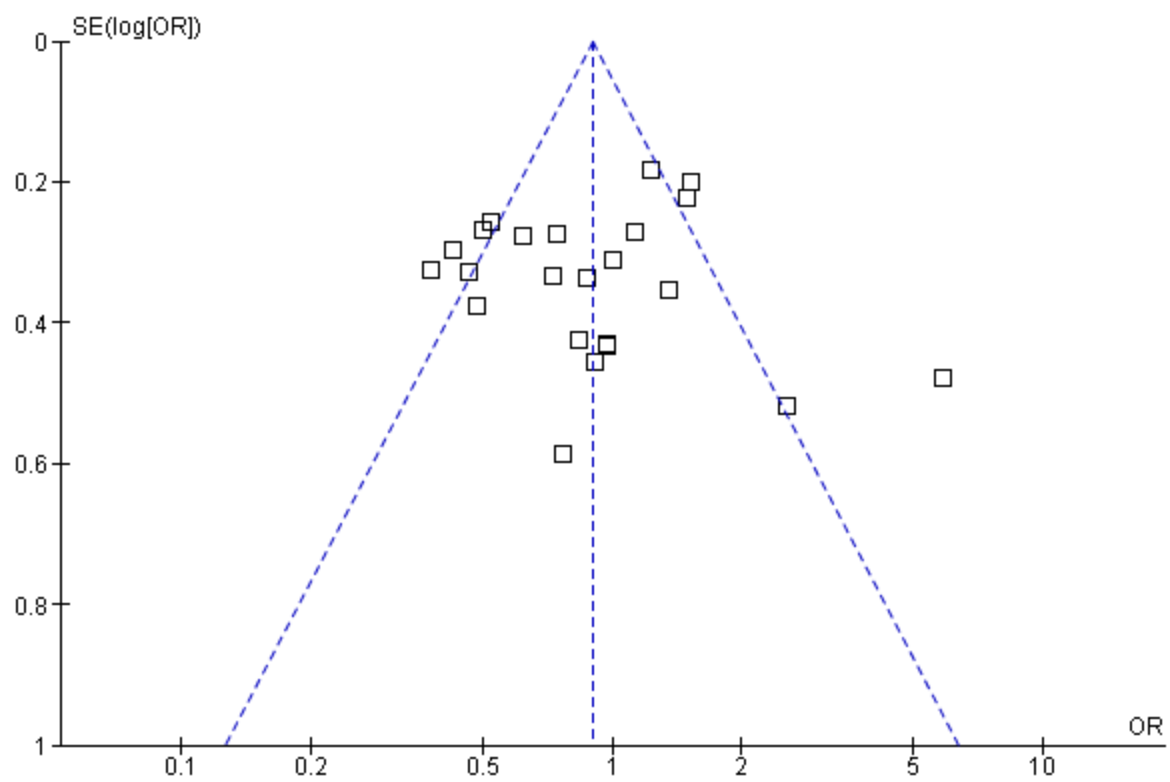

Supplement: Additional file 3 — Figure S1. Funnel plot of CD14 -260 (-159) TT versus CC genotypes for all reviewed studies. Standard error of the logarithm of the odds ratio (SE(log[OR])) was plotted against the OR of each study. [file 1471-2350-12-93-S3.PDF]

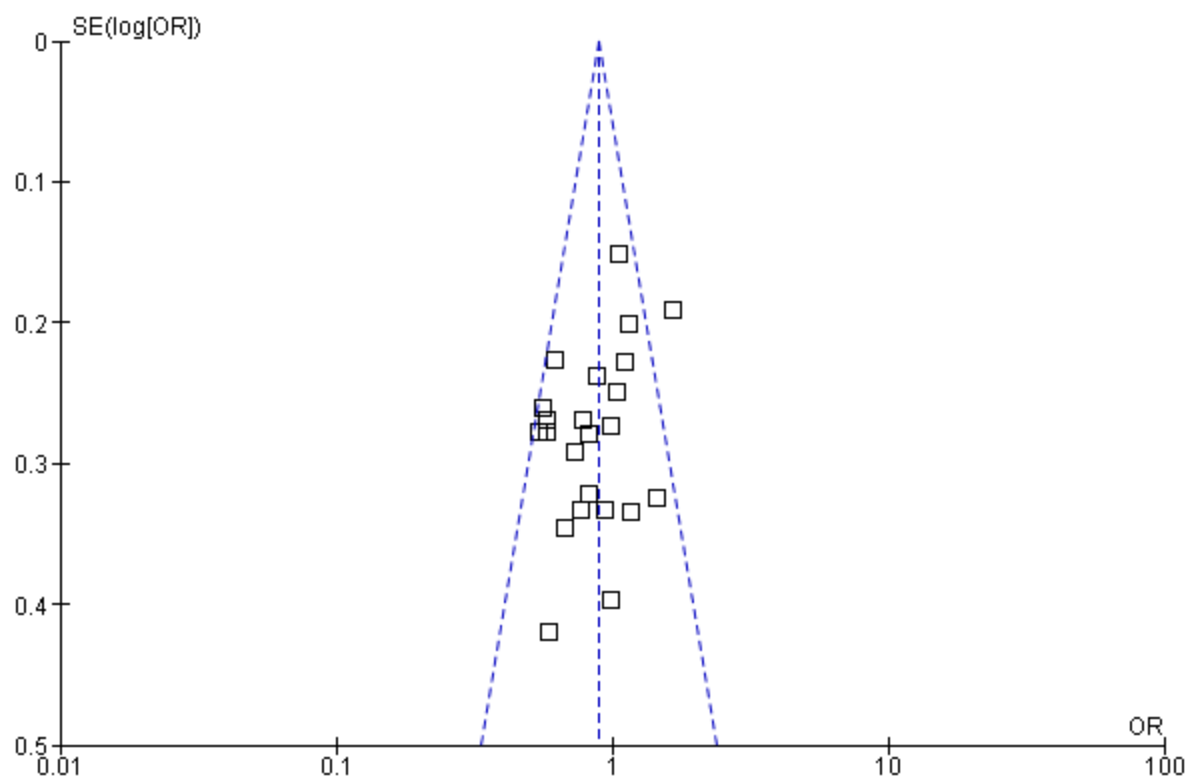

Supplement: Additional file 4 — Figure S2. Funnel plot of CD14 -260 (-159) CT versus CC genotypes for all reviewed studies. Standard error of the logarithm of the odds ratio (SE(log[OR])) was plotted against the OR of each study. [file 1471-2350-12-93-S4.PDF]

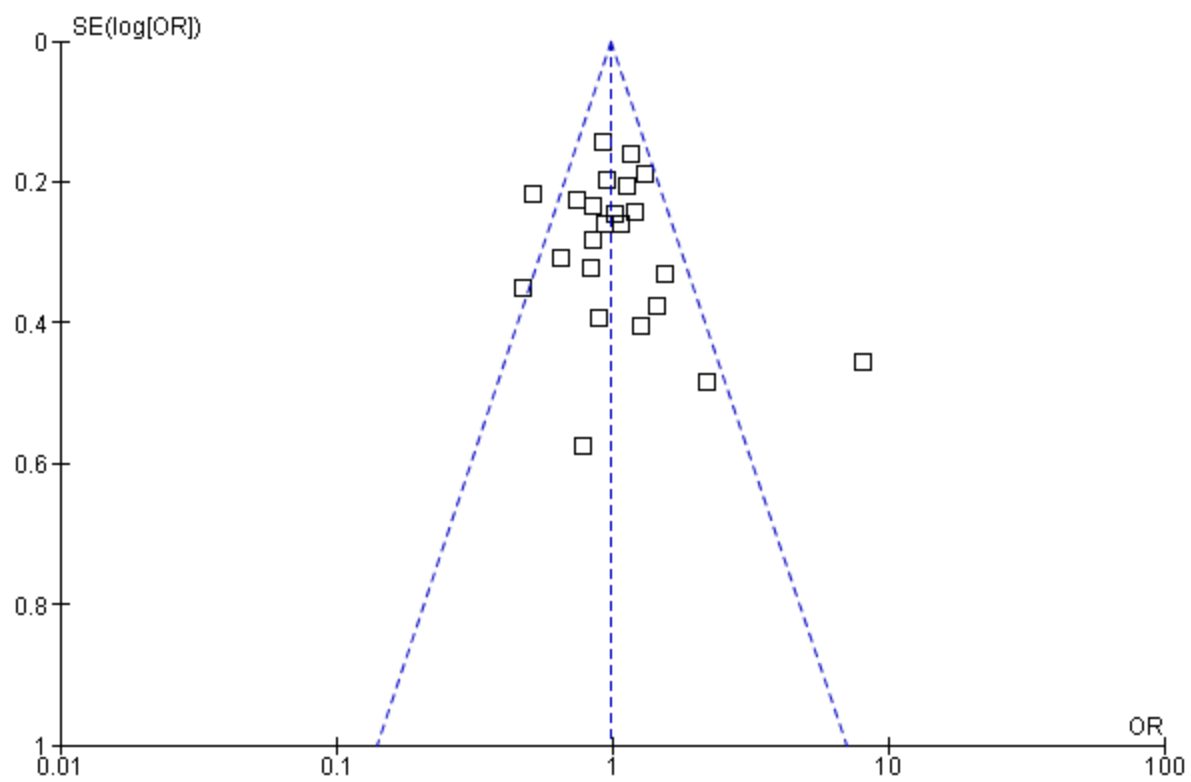

Supplement: Additional file 5 — Figure S3. Funnel plot of CD14 -260 (-159) TT versus CT genotypes for all reviewed studies. Standard error of the logarithm of the odds ratio (SE(log[OR])) was plotted against the OR of each study. [file 1471-2350-12-93-S5.PDF]

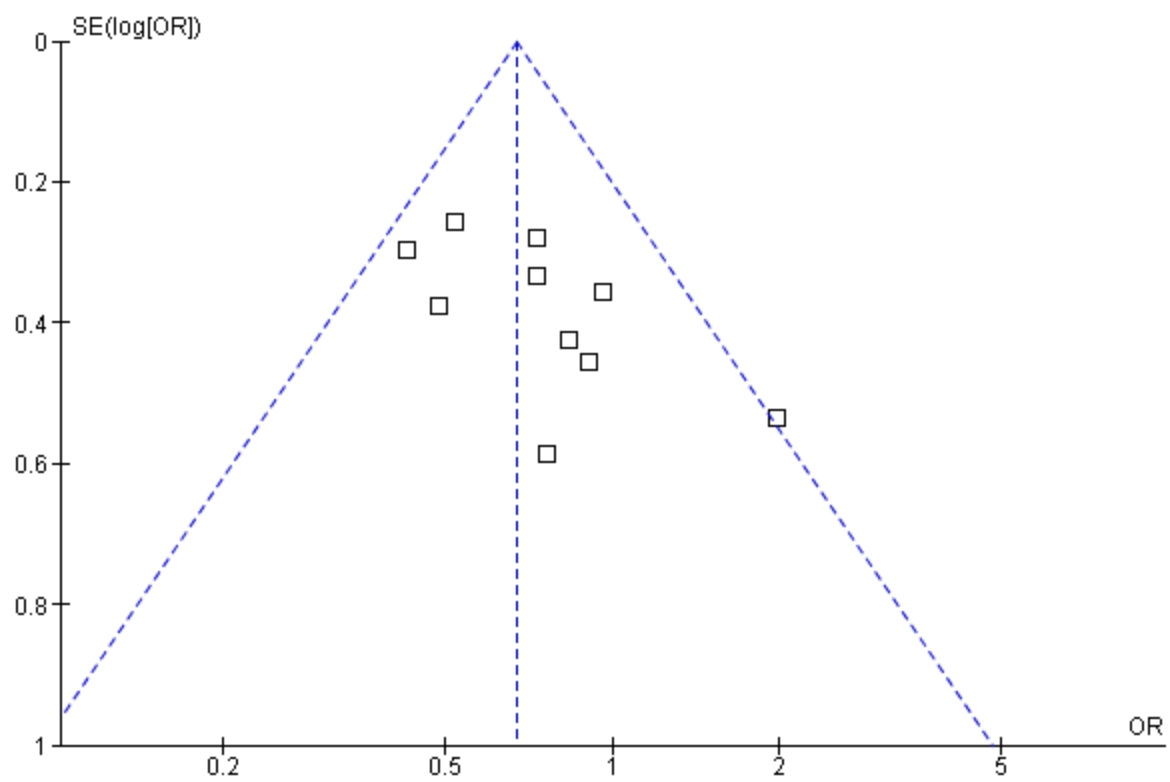

Supplement: Additional file 6 — Figure S4. Funnel plot of CD14 -260 (-159) TT versus CC genotypes for studies with precisely defined phenotypes. The funnel plot displays studies included in the review that used atopic asthma cases and non-atopic non-asthmatic controls, excluding heterogeneous studies identified by sequential analysis [11,27,42]. Standard error of the logarithm of the odds ratio (SE(log[OR])) was plotted against the OR of each study. [file 1471-2350-12-93-S6.PDF]

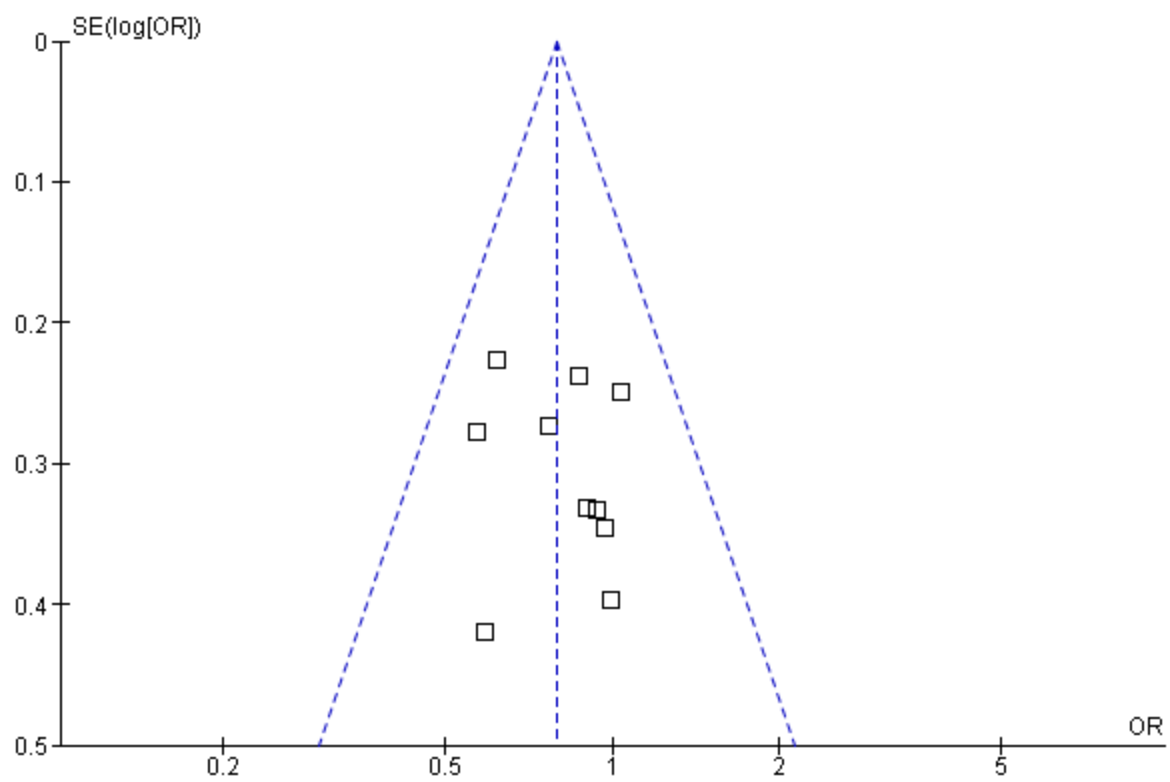

Supplement: Additional file 7 — Figure S5. Funnel plot of CD14 -260 (-159) CT versus CC genotypes for studies with precisely defined phenotypes. The funnel plot displays studies included in the review that used atopic asthma cases and non-atopic non-asthmatic controls, excluding heterogeneous studies identified by sequential analysis [11,27,42]. Standard error of the logarithm of the odds ratio (SE(log[OR])) was plotted against the OR of each study. [file 1471-2350-12-93-S7.PDF]
